# Development of a Hepatoprotective Herbal Drug from *Turnera diffusa*

**DOI:** 10.1155/2022/5114948

**Published:** 2022-01-10

**Authors:** Cecilia Delgado-Montemayor, Paula Cordero-Pérez, Liliana Torres-González, María de la L. Salazar-Cavazos, Alma L. Saucedo, David Paniagua-Vega, Noemí H. Waksman-Minsky

**Affiliations:** ^1^Universidad Autonoma de Nuevo Leon, Departamento de Química Analítica, Facultad de Medicina, Monterrey 64460, Mexico; ^2^Universidad Autonoma de Nuevo Leon, Unidad de Hígado, Hospital Universitario “Dr. José Eleuterio González”, Monterrey 64460, Mexico; ^3^Consejo Nacional de Ciencia y Tecnología (CONACYT), Ciudad de México 03940, Mexico

## Abstract

The incidence of liver diseases, such as nonalcoholic fatty liver disease and drug-induced liver injury, continues to rise and is one of the leading causes of acute hepatitis. Current trends suggest that these types of conditions will increase in the coming years. There are few drugs available for the prevention or treatment of hepatic diseases, and there is a growing need for the development of safe hepatoprotective agents. The medicinal plant, *Turnera diffusa*, has many ethnopharmacological uses, one of which is the production of a flavonoid named hepatodamianol, which is the principal component responsible for this plant's hepatoprotective properties. In the present study, we describe the development and standardization of an active extract obtained from *T. diffusa*. We conducted nuclear magnetic resonance spectroscopy to identify hepatodamianol unambiguously in each sample. Using this extract, hepatoprotection could be demonstrated *in vivo* for the first time. The hepatoprotective effect did not display a significant difference *in vivo* when compared with silymarin used as a positive control at the same doses. Implementation of quality criteria used for standardization, such as flavonoid and hepatodamianol content, hepatoprotective activity, and absence of residual solvents, will allow future preclinical trials with this herbal drug.

## 1. Introduction

The liver is the most metabolically active organ of the body and is responsible for the detoxification and deposition of endogenous and exogenous compounds [1, 2]. This fact makes the liver susceptible to injury and the possible development of acute or chronic diseases. Several metabolic disorders are associated with significant hepatotoxicity and can even lead to death. Major risk factors pertinent to cirrhosis, steatohepatitis, hepatitis, and hepatotoxicity include xenobiotics, diabetes, obesity, pollutants, free radicals, food additives, and alcohol [3, 4]. Liver diseases remain one of the most severe health problems, and their management is still a challenge to current medical care. The most prevalent causes of liver disease are those associated with alcohol, nonalcoholic fatty liver disease, and hepatitis C/B virus. Oxidative stress has been implicated in the pathogenesis of liver disease; free radicals have been reported to cause oxidative stress and can be blocked by antioxidant hepatoprotective agents [5, 6]. According to the World Health Organization, liver diseases affect 10% of the world's population, with around 2.4 million deaths annually associated with liver disease [7]. In México, hepatic illnesses were the fourth leading cause of mortality during 2018 [8].

**Figure 1:**
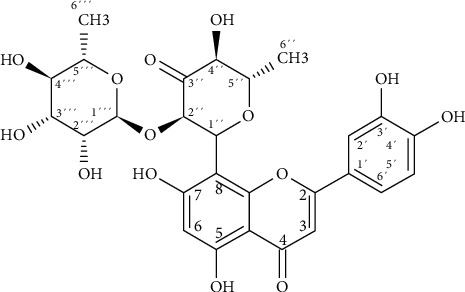
Hepatodamianol structure.

There are few drugs available for the prevention or treatment of hepatic diseases, and there is a growing need for the development of safe hepatoprotective agents. While many different proposals aim to discover new medicines, natural products remain one of the best reservoirs of novel bioactive chemical structures. Almost 70% of new chemical prototypes for designing medicines over the past nearly four decades have been obtained or inspired by natural product sources [9]. México possesses one of the most extensive regions of botanical diversity on Earth, with more than 4,000 plants used for medicinal purposes. Several different plants have traditionally been used to treat liver diseases in México [10]. In a preliminary survey of medicinal plants from northeast México, we demonstrated the hepatoprotective effect of a methanolic extract of the aerial part of *Turnera diffusa* using an *in vitro* model based on the induction of damage by carbon tetrachloride (CCl_4_) [11]. More recently, through a bioassay-guided fractionation, one of the main bioactive metabolites, named hepatodamianol, was identified (Figure 1) [12]. The hepatoprotective effect of hepatodamianol was four times higher in an *in vitro* test than the positive control, silibinin [13].

Recent results suggest that a methanolic extract obtained from *T. diffusa* shows an antifibrotic effect by decreasing profibrotic and mitochondrial markers together with the possible induction of apoptosis through transcription factor SNAI1 expression in activated human hepatic stellate cells [14].

We visualized a proposal to produce a standardized extract from *T. diffusa* because of the complicated and expensive process of hepatodamianol isolation and purification. Therefore, the current study presents the development, standardization, and evaluation of an active extract obtained from *T. diffusa*. Using this plant extract, *in vivo* hepatoprotection could be demonstrated for the first time. Quality criteria will allow standardization of this extract for future preclinical trials with this herbal drug.

## 2. Materials and Methods

### 2.1. Materials

All solvents were of analytical grade and purchased from Fermont (Monterrey, Nuevo León, México), except for HPLC-grade methanol (MeOH) purchased from JT Baker (Fisher Scientific, Fair Lawn, NJ, USA) and hexadeuterodimethyl sulfoxide (DMSO-*d*_6_, 99.8%D) purchased from Sigma-Aldrich Chemical Co. (St. Louis, MO, USA). MilliQ deionized water was obtained with an Elga II MilliQ system (Veolia, México). The following reagents were purchased directly from Sigma-Aldrich Chemical Co.: 2,2-diphenyl-1-picrylhydrazyl (DPPH), aluminum trichloride (AlCl_3_), quercetin, rutin, silibinin, silymarin, CCl_4_, and 3-(4,5-Dimethyl-2-thiazolyl)-2,5-diphenyl-2H-tetrazolium bromide (MTT), and trypan blue. Dimethyl sulfoxide (DMSO) was purchased from ACS Research Organics (Cleveland, OH, USA). Dulbecco's modified Eagle's medium (DMEM), fetal bovine serum (FBS), trypsin-EDTA 0.25% (1x), penicillin G (100 IU/mL), streptomycin (100 *μ*g/mL), and phosphate-buffered saline (PBS) were purchased from Gibco Invitrogen (Carlsbad, CA, USA).

### 2.2. Plant Material

The aerial part of *T. diffusa* was collected on 10 different dates and in 10 different locations in Nuevo León, México, during 2014–2016. The collective specimens were dried at room temperature, ground finely, and stored in a dry and cool place until use. The plant was authenticated by the Institutional Herbarium at the Biology School of the Universidad Autónoma de Nuevo León, and a voucher specimen (No. 23,569) was deposited therein.

### 2.3. Optimization of the Extraction Method

The powdered plant (500 g) was extracted by maceration with MeOH in a shaker stirring at room temperature. The supernatant was filtered and evaporated under reduced pressure at 37°C, dried, and stored at 4°C in an oxygen-free environment. For the optimization, a full factorial design experiment of four factors (solvent volume, number of extractions, time, and stirring speed), two levels (lower and higher), and two responses (overall yield and hepatodamianol amount) were performed (Modde software version 8.0, UMETRICS AB, Umeå, Sweden). The coefficient graphs were generated with the results of the factorial design using the multiple linear regression (MLR) method. The coefficient size represents the change in the response when a factor changes its value from 0 to 1 in coded units. The effect of the variable was considered significant when the confidence interval did not cross zero.

### 2.4. Fractionation

From the methanolic extract (ME) obtained using the optimized procedure, we followed the fractionation previously described to obtain hepatodamianol. Only the active fractions were further tested. The chlorophylls were eliminated using SEP-PAK C-18 cartridges (1000 mg/8 mL; Alltech Associates Inc., Deerfield, IL, US); the samples were eluted with 70 mL of 50%, 70%, and 100% MeOH. The fraction eluted with 50% MeOH was named CME. Three grams of CME was subjected to silica vacuum liquid chromatography using CH_2_Cl_2_, AcOEt, AcOEt : MeOH (1 : 1), and MeOH as eluents (200 mL of each solvent). The fraction eluted with AcOEt : MeOH (1 : 1) was named FrVLC. This fraction was further separated by low-pressure reverse phase chromatography in a C-18 column (40–63 *μ*m, Licoprep); 400 mg of FrVLC were eluted with MeOH 40, 50, 60, and 100% (300 mL each). The fraction obtained with MeOH 60% was named flavonoid mixture (FrMF).

### 2.5. In Vitro Cell Test

Cell culture and viability determination: cells were grown in 75 cm^2^ flasks with DMEM Advanced medium, supplemented with 10% FBS, 1% L-Glutamine, and 1% of antibiotics (penicillin and streptomycin), at 37°C in a 5% CO_2_ humidified atmosphere. These conditions were the same for all cell tests. When cells reached 70–80% confluency, they were washed with PBS (2 ∗ 10 mL), and trypsin-EDTA was added (1 mL 0.25%, 5 min). Once the cells were in suspension, they were counted after staining with trypan blue as a viability indicator.

#### 2.5.1. Cytotoxicity Test

MTT assay was performed to measure the cytotoxic effect of the Vero cell extracts (ATTC CCL-81, normal kidney epithelial cell extracts from African green monkey) [15]. The cells were seeded in 96-well plates (7 × 10^3^ cells per well) and incubated at 37°C with 5% CO_2_ for 24 hours before carrying out the assays. The cells were treated with different concentrations of the extracts (500 to 4 *μ*g/mL) and doxorubicin (50 to 0.4 *μ*g/mL). After 48 h of incubation, the treated cells were washed with PBS buffer twice; subsequently, 150 *μ*L of 0.5 mg/ml of MTT were added to each well and incubated for an additional 3 h. After that time, the supernatant was decanted, and 200 *μ*L of DMSO was added to each well. The absorbance was read at a wavelength of 540 nm using a microplate reader (Multiskan FC, Thermo Scientific Inc., Waltham, Massachusetts, USA), and the CC_50_ value was calculated. Doxorubicin was used as a cytotoxicity control.

#### 2.5.2. Hepatoprotection Test

HepG2 cells (hepatoma cell line, ATCC HB-8065) were used for the hepatoprotection test. The cells were then seeded in 6-well plates (1 million cells per well) and incubated for 24 hours before carrying out the assays. The protective effect against CCl_4_ in HepG2 cells was assessed after preexposure to silibinin (positive control for hepatoprotection, 100 *μ*g/mL, for 1 h) or fractions obtained from *T. diffusa* (100 *μ*g/mL, for 1 h). The medium and extract were removed, and the toxicity was induced by adding CCl_4_ (0.4%, for 2 h). The cellular viability was evaluated by the measurement of aspartate aminotransferase (AST) released into the medium [16]. AST levels were quantified using the ILab Aries instrument (Instrumentation Laboratory SpA, Milan, Italy).

### 2.6. Quality Control

#### 2.6.1. Antioxidant Activity

The antioxidant activities were determined by the DPPH assay, as described by Granados-Guzmán et al. [17]. Solutions of each extract/fraction (1 mg/mL) and positive control (quercetin 100 *μ*g/mL) were prepared in ethanol (EtOH). 100 *μ*L was added to the first well of a polystyrene 96-well plate containing 100 *μ*L EtOH. From this well, a serial dilution with EtOH (factor 1 : 2) was made. Then, 100 *μ*L DPPH 280 *μ*M was added to each well; and the mixtures were incubated in the dark for 15 min at room temperature. Absorbance was recorded with a microplate reader at 517 nm using EtOH as the blank. The reduction percentage of the light-absorbing species was calculated in the absence of an antiradical agent (negative control) and the presence of an antiradical agent using

(1) $$\begin{array}{c} \%\text{ reduction}=\left[\frac{A-B}{A}\right]*100, \end{array}$$

where *A* is the absorbance of the blank and *B* is the absorbance of samples. A linear regression curve with the percentage reduction as a function of the concentration of each sample was generated, and the concentration that effectively reduced DPPH by 50% (EC_50_) was calculated. All experiments were performed in triplicate, and the mean and standard deviation (SD) were calculated in each case.

#### 2.6.2. Determination of Total Flavonoids

The total flavonoid content was carried out as described by Kumazawa et al. [18]. Briefly, 100 *μ*L of each sample (1 mg/mL in EtOH) was added to the first well of a polystyrene 96-well plate containing 100 *μ*L EtOH. From this well, a serial dilution with EtOH (factor 1 : 2) was made. One hundred *μ*L AlCl_3_ (10%) was added to each well and left in the dark for 1 h. After this time, absorbance was recorded with a microplate reader at 510 nm using EtOH as the blank. Quercetin and rutin were used as positive controls. Total flavonoid content was calculated using

(2) $$\begin{array}{c} \frac{\text{mg flavonoid}}{\text{g sample}}=\left[\frac{A-b}{m}\right]*1000, \end{array}$$

where *A* is the absorbance of the sample, *b* is the “*y* intercept,” and *m* the slope of the calibration curve. The result was expressed as mg flavonoids equivalent to quercetin or rutin per g of the sample obtained from a respective calibration curve. All experiments were conducted in triplicate.

#### 2.6.3. Residual Solvent Determination

The presence of residual solvent was detected by GC/MS in an Agilent Technologies 6890N chromatographer coupled to a mass spectrometer 5973 INERT (Santa Clara, California, USA), using a capillary column DB-WAX (30 m, 0.25 mm internal diameter). Conditions were as those of the 467 USP Method (USP 32-NF 27) [19]. Ten milligrams of the lyophilized samples was analyzed employing headspace as indicated by the 467 USP method.

#### 2.6.4. Quantification of Hepatodamianol by HPLC-DAD

HPLC-DAD was performed using a Waters Alliance 1525 liquid chromatography system equipped with an online degasser, binary pump, autosampler, and a 2996 diode array detector (Waters, Milford, Massachusetts, USA). The separation was carried out in an inverse phase Luna Phenomenex column (C-18, 150 × 4.6 mm, 5 *μ*m) with a Phenomenex precolumn (C-18 4 × 3.0 mm). The mobile phase consisted of a gradient of (A) MeOH and (B) formic acid 0.1%, elution starting with 30% A; 30–60% A, 0–20 min; 60–70% A, 20–25 min; and 70% A, 25–60 min. The flow rate was 0.4 mL/min; 0–20 min, 0.2 mL/min; 20–45 min; 0.4 mL/min, and 45–60 min. Injection volumes were 20 *μ*L. All samples were injected in duplicate.

### 2.7. Identification by Nuclear Magnetic Resonance Spectroscopy (NMR)

NMR experiments were recorded on a 400 MHz Avance III (Bruker, Karlsruhe, Germany) spectrometer equipped with a 5 mm Broadband Observe (BBO) probe with a z-gradient pulse field. Samples of the selected extracts (50 mg) were dissolved in 600 *μ*L DMSO-*d*_*6*_, sonicated, and centrifuged (13000 ×g, 5 min at room temperature). Finally, 600 *μ*L of the supernatant was transferred to standard 5 mm NMR tubes. ^1^H-NMR spectra were recorded using a 30° pulse experiment, under the following acquisition parameters: 16 and 128 scans with a fixed receiver gain value of 32, the spectral width of 20.0 ppm, 65,536 points in the time domain, and acquisition time 4.0 s. Selective total correlation spectroscopy spectrum (1D-TOCSY) was used to identify hepatodamianol in each sample, as previously described by Lucio-Gutiérrez et al. [20]. 1D-TOCSY lselmlpg pulse sequence was used. Data were collected in DQD acquisition mode with 65,536 points in the time domain, 128 scans, and a 20.0 ppm spectral width. The transmitter frequency offset for channel F1 (O1) was fixed according to the resonance frequency of the selected signals: 6´´´ at 209.17 Hz (0.512 ppm) and 6´´ at 564.84 Hz (1.402 ppm), respectively. Mixing time (D9) was evaluated between 20 and 200 ms to optimize magnetization transfer to the whole spin system. In addition, hepatodamianol resonances assignment in DMSO-*d6* was confirmed by selective 2D NMR COSY, TOCSY, HSQC, and HMBC data. All experiments were acquired at 298 K, and the residual protonated solvent signal was used to reference chemical shifts (DMSO-*d*_*5*_, 2.500 ppm). NMR data were manually processed and analyzed with Top Spin 3.6 software (Bruker Company, Billerica, Massachusetts, USA).

### 2.8. Chemometric Analysis

MATLAB R2015a software (MathWorks Inc., Massachusetts, USA) and PLS_Toolbox software (Eigenvector Research, Inc., Washington, USA) were used to conduct a multivariate analysis of the processed NMR data. Principal component analysis (PCA) was performed using all the signals obtained between 0 and 14 ppm, excluding the regions where solvent resonances appear. The first and second components were used with a confidence level of 95%. Correlation optimized warping (COW) was performed for optimized alignment. The maximum cumulative product of the correlation coefficients was used to find the sample that displayed the highest similarity to the rest. This one was considered the most representative of the whole.

### 2.9. In Vivo Hepatoprotection Test

The animal procedures were performed according to the specifications of the Official Mexican Norm NOM-062-ZOO-1999. This project was approved by the Ethics and Research Committee of the School of Medicine, Universidad Autónoma de Nuevo León. Assays were conducted as described by Cordero-Pérez et al. [21]. Briefly, female Wistar rats (200–300 g) were housed under standard laboratory conditions (24 ± 3°C) on a 12 h dark and light cycle, fed with standard commercial pellets (Nutrimix de México, S.A. de C.V., México City, México) and water *ad libitum.* The rats were randomly divided into 4 groups of 4 animals each. Group I (negative control) and group II (damage control) received distilled water (1 mL, orally). Group III (hepatoprotection control) received silymarin 70 mg/kg (in a maximum 1 mL volume), and group IV (experimental group) received the herbal drug (HM) 70 mg/kg of the sample (in maximum 1 mL). All the groups received their treatment for 3 days orally every 12 hours. A blood sample was taken before the first administration and after the second delivery on the third day. For the liver damage, CCl_4_ (1 ml/kg) 50% in mineral oil was administered intraperitoneal to groups II, III, and IV. Group I received only mineral oil. After 24 h of damage induction, rats were anesthetized by intraperitoneal injection with 100 mg/kg of ketamine (Anesket, PiSA Agropecuario, S.A. de C.V. Reg SAGARPA Q7833-028, Guadalajara, Jal, México) and 10 mg/kg of xylazine (Sedaject, Vedilab S.A. de C.V. Reg. SAGARPA, Q-0088-122, Querétaro, Qro., México). After anesthesia, rats were shaved and asepsis of the abdominal region was performed using Microdacyn antiseptic solution (Oculus Technologies of México, S.A. de C.V. Guadalajara, Jal., México) followed by a 20% solution of chlorhexidine gluconate (Farmacéuticos Altamirano de México, S.A. de C.V., México City, México). A midline incision was then performed, and 5 mL of blood was withdrawn by cava vein phlebotomy, causing death by exsanguination. Serum was separated from blood samples by centrifugation at 2,000 g for 12 min and stored at –80°C until use. AST and alanine aminotransferase (ALT) levels were quantified in the ILab Aries instrument (Instrumentation Laboratory SpA, Milan, Italy). Data were analyzed using a one-way analysis of variance followed by the Tukey *post hoc* test. The analysis was performed using GraphPad software version 8.0. The results are expressed as mean ± SD. Differences between means were considered significant at *P* < 0.05.

## 3. Results and Discussion

Previously, we demonstrated the *in vitro* hepatoprotective effect of both ME obtained from *T. diffusa* and hepatodamianol and C-glycoside isolated for this plant [10, 13]. To carry out *in vivo* studies with hepatodamianol, it is necessary to have a sufficient quantity of the compound in a considerable degree of purity, which means high costs and time for its purification. For this reason, we proposed in this study to standardize an extract from the plant, enrichened in hepatodamianol, and use it to carry out hepatoprotection tests *in vivo*. The evaluation of a standardized extract has the additional advantage in that it will allow preclinical and clinical tests to be carried out in the future.

### 3.1. Fractionation of the Methanolic Extract

The first step was to define which was the best option for this goal if the crude extract or any active fraction was obtained from it. We followed the procedure previously described to purify hepatodamianol to isolate an active fraction that did not require numerous and expensive steps. In addition, *In vitro* hepatoprotection and cytotoxicity were tested. Results are shown in Table 1.

**Figure 2:**
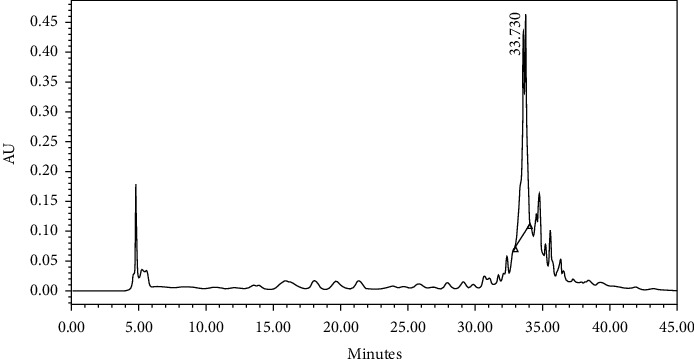
Chromatogram obtained of FrVLC, RP C-18 HPLC-DAD, and wavelength 254 nm. As can be seen, several compounds coelute in this fraction. However, the higher intensity peak corresponds to hepatodamianol at 33.73 min.

After comparing the results shown in Table 1, we can observe that although the ME is slightly cytotoxic, toxicity is lost during the fractionation process, making the successive fractions less cytotoxic. Cytotoxicity of ME from *T. diffusa* in different cell lines was previously reported by Avelino-Flores et al. [22] and Willer et al. [23]. Although FrMF is more active than FrVLC, FrMF is obtained after a subsequent step of chromatographic purification, increasing costs and process time and decreasing the yield of enriched extract. Furthermore, the difference in hepatoprotection activity between these two fractions is not significant. Therefore, we selected FrVLC as the best fraction to obtain the standardized extract. As can be seen in the chromatograms shown (Figure 2), this fraction is enriched in hepatodamianol.

### 3.2. Optimization of the Extraction Method

Once we selected the fraction to be used, we conducted an optimization of the extraction steps. Solvent volume, number of extractions, time, and stirring speed were considered as variables. A complete factorial design with 19 experiments (16 designs and 3 central values) was conducted. The responses were percent recovery and hepatodamianol content.  Table 2 shows the variables and levels tested.

**Figure 3:**
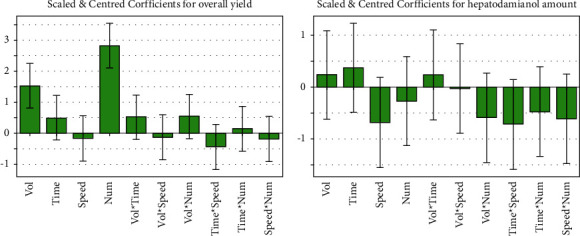
Coefficient plots of the responses obtained with the experimental design used to optimize the extraction, obtained by means of MLR. (a) Effect of the variables on recoveries. (b) Effect of the variables on hepatodamianol content. Variables under evaluation: solvent volume, time, speed, and the number of extractions.

The results obtained were analyzed employing MLR to generate coefficient graphs. We observed that no variable or combination of variables significantly affected the variable, “hepatodamianol quantity” (*P* < 0.05). On the contrary, the volume of solvent and number of extractions significantly affected the “recovery” (*P* < 0.05). The other factors were considered, and their interaction did not significantly affect the results (Figure 3).

To carry out the optimization of the variables, we selected the experiment that yielded the highest recoveries (25 mL, 2 h stirring, 100 rpm, and 6 extractions). We performed independent experiments for each one of the variables under consideration. One of these was the solvent volume used for each extraction. We kept the total volume of solvent constant (at 150 mL) and fractionated it into 1, 3, 4, and 5 extractions. To assess the influence of the other variable under consideration, the number of extractions, we tested volumes of 15, 20, and 25 mL MeOH using, for each volume, 3, 4, and 5 extractions.

The results showed that the optimal conditions for the highest recovery (10.4%) were 3 successive extractions, 50 mL MeOH, and 2 h stirring at 300 rpm. Therefore, 10 different samples collected at different times were extracted using the optimized extraction procedure. Each one was further fractionated to obtain the FrVLC. These samples were named HM1 to HM10.

### 3.3. Quality Control

To verify the purity, identity, and activity of the different HM obtained, some parameters were assessed.  Table 3 shows the results obtained for each sample.

Hepatodamianol was identified and quantified by HPLC-DAD. As expected, all samples presented similar chromatographic patterns. The hepatodamianol content in the different samples was between 7.91 and 25.73 mg per g sample.

All samples showed high flavonoid content and antioxidant activity as well. However, HM9 was by far the sample that presented the highest content in flavonoids, hepatodamianol, and the most considerable antioxidant activity. All the samples showed hepatoprotection *in vitro*, and despite the differences found in flavonoid and hepatodamianol content between the samples, the hepatoprotection *in vitro* did not show significant differences (ANOVA *P* > 0.05). Interestingly, the hepatoprotective activity of all the extracts tested was better than the activity of silibinin used as a positive control, a well-known hepatoprotective molecule [24]. With these results in mind, it is likely that while hepatodamianol is one of the compounds responsible for the hepatoprotective activity in *T. diffusa* extracts, the synergy between all the components plays an essential role in the overall activity. For this reason, although hepatodamianol has been considered a biomarker of this plant, it will be interesting to know the structure of the minority flavonoids present in these extracts [25].

Biomarkers can be identified using selective experiments that allow isolation of the signal(s) belonging to a particular compound through scalar or dipolar coupling. Among them, a selective 1D-TOCSY experiment offers the advantage of establishing ^1^H connectivity through *J*-coupling in a spin system. This kind of “spectroscopic isolation,” also known as spin chromatography, is useful for generating an isolated spectroscopic pattern of the irradiated spin system to overcome some of the problems associated with the signal overlapping. In fact, 1D-TOCSY has been considered a type of chromatographic spectroscopy [26, 27]. Papaemmanouil et al. [26] introduced the term “NMR spin-chromatography” to emphasize the advantages of the selective 1D-TOCSY experiment in the analysis of complex mixtures through the selective excitation of nuclei, without the necessity of previous isolation steps. Recently, we reported applying the selective 1D-TOCSY experiment for the unambiguous identification of hepatodamianol in crude extracts of *T. diffusa* as a strategy to verify the authenticity of *T. diffuse* botanical material in combination with chemometrics methods [20]. The selective 1D-TOCSY experiments of the rhamnopyranosyl and xylo-hexopyranos-3-uloside moieties were obtained by specific irradiation on the resonance frequencies of 6´´ and 6´´´ methyl groups at 0.512 and 1.401 ppm, respectively. NMR signals from both motifs provide a spectroscopic fingerprint for hepatodamianol (Supplementary Material, Figures S1 and S2).

As a purity parameter, residual solvent content was analyzed by headspace CG/MS. Lyophilized samples of the HM did not present any residual solvent (data not shown).

**Figure 4:**
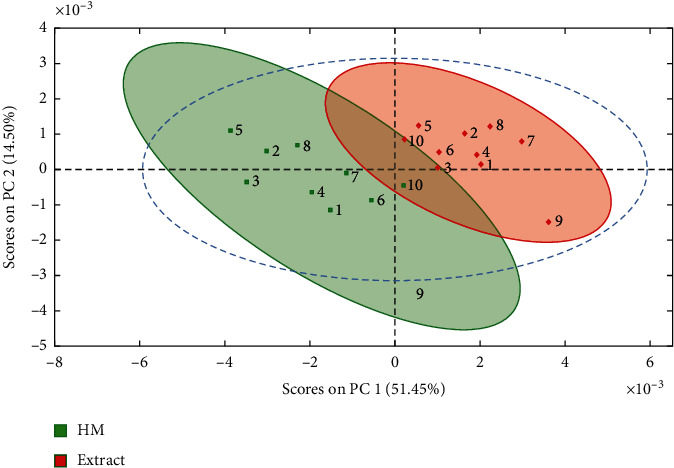
PCA analysis of ^1^H-NMR data of extracts (red) and HM (green) of *T. diffusa* samples. The shadowed areas indicate the Hotelling ellipse (95%). As can be seen, in both groups, sample 9 is far from their respective cluster, and in the case of HM samples, it is outside of the confidence area delimited by the dashed line. This statistical deviation of HM9 is related to variations in the resonance patterns detected in the NMR spectra.

**Figure 5:**
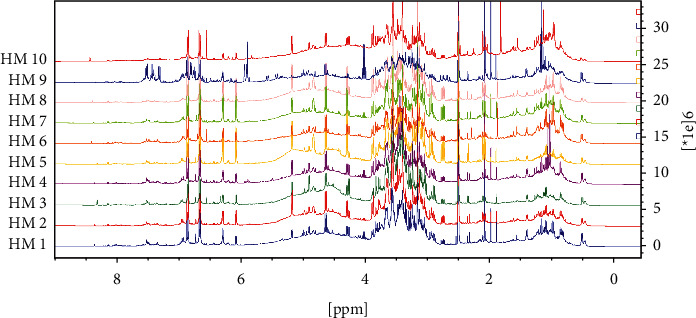
^1^H-NMR spectra of HM1-HM10 samples, 400 MHz, DMSO-*d*6. In this stacked view, a close similarity in chemical composition between samples is observed, except for HM9. In this sample, a remarked difference can be noticed in the aromatic region (5.9–7.8 ppm), where strong resonances related to flavonoids were detected.

Further, NMR data obtained for HM were compared using multivariate statistics to achieve an integral analysis of the results. ^1^H-NMR spectra were used for this purpose because this technique allows the observation of signals among a mix of compounds present in the extracts. Moreover, the analysis is simultaneous, which allows a global characterization of the sample. PCA analysis (Figure 4) showed that HM9 does not group with the rest of the samples. ^1^H-NMR obtained from HM9 shows strong signals at 4, 5.9, and 7 ppm that were not observed in the rest of the samples (Figure 5).

From the analysis of the graphic loads for PC1 and PC2, we found that the signals in the aromatic region were responsible for this clustering. However, in both cases, it seems that HM9 does not group with the rest of the samples. This suggests that HM9 has a different flavonoids content from the rest of the samples.

With the data obtained from ^1^H-RMN of the samples (excluding HM9), a COW was performed to optimize the alignment, and the maximum cumulative product of the correlation coefficients was employed to find the sample that displayed the highest similarity to the rest. HM4 showed the highest preponderance, and therefore, that sample was selected as the most representative to conduct *in vivo* tests.

#### 3.3.1. In Vivo Hepatoprotection Test

**Figure 6:**
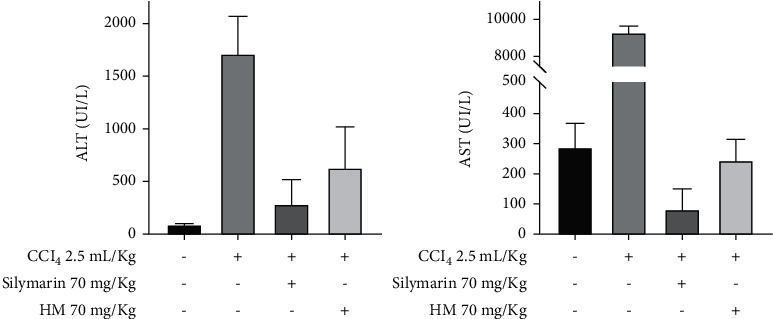
Hepatoprotection *in vivo*, AST and ALT levels in serum. *n* = 4. Data are expressed as mean ± standard deviation. MH4 as the HM tested. ^*∗*^Statistically similar (Student's *t*-tests), *P* > 0.05. ^*∗∗*^Statistically different, *P* < 0.05, with respect to the HM.

To conduct the *in vivo* hepatoprotection analysis, we followed the model reported by Cordero Pérez et al. [21]. Serum activities of AST and ALT were measured. Levels found for both enzymes were significantly lower (*P* < 0.02) when compared with the CCl_4_ group (damage control). Levels of ALT between the test group and the silymarin group (hepatoprotection group) were not significantly different (Figure 6). In contrast, AST levels differed between these two groups (*P* < 0.05), being more active in the silymarin group. Nevertheless, enzymatic levels found between the negative control and the test group did not show any significant differences (Figure 6). Therefore, we consider that the standardized extract we obtained from *T. diffusa* is a potential hepatoprotective agent with an effect similar to that of silymarin in the *in vivo* model used by us.

Recent results suggest that the ME obtained from *T. diffusa* shows an antifibrotic effect by decreasing profibrotic and mitochondrial markers together with the possible induction of apoptosis through transcription factor SNAI1 expression in activated human hepatic stellate cells [14].

Brito et al. [28] reported hepatoprotective activity *in vivo* of a ME obtained from *T. ulmifolia* using a CCl_4_ damage agent model. Their results are very similar to the results with *T. diffusa* extract reported here. However, the doses used by Brito et al. [28] were different. CCl_4_ was administered at 2.5 mL/kg diluted 50% v/v in corn oil. That is, the hepatotoxic agent was 2.5 times more concentrated than in our experiments. Furthermore, silymarin was administered at 50 mg/kg and the extract at 500 mg/kg. In the present study, we used the same concentration both for the positive drug (silymarin) and for the extract under examination, 70 mg/kg. Therefore, although our results are similar to those obtained by Brito et al. [28], we are unwilling to compare, considering the differences in doses used.

## 4. Conclusions

In conclusion, our experimental findings show for the first time the hepatoprotective effect *in vivo* of a standardized extract obtained from *T. diffusa*. Through experimental design, we optimized the extraction step and designed a standardized procedure for the fractionation of *T. diffusa*, which was applied to 10 different authenticated samples. All the samples obtained contained hepatodamianol and showed hepatoprotection *in vitro*, in a manner similar to the activity shown by silymarin (positive control). Hepatodamianol in all the samples was identified unequivocally using HPLC/DAD and ^1^H-NMR and selective 1D-TOCSY. Using proton NMR data, we performed multivariate analysis to demonstrate the chemical similarity in extracts and select the most representative samples to conduct *in vivo* assays. *In vivo* hepatoprotective effect of the standardized extract from *T. diffusa* showed no significant difference compared with the activity displayed by silymarin at the same doses. These results encourage us to pursue further research to develop this extract as a phytomedicine. For this purpose, we find it desirable to identify the minor flavonoids present in the extracts.

Experiments to further explore the mechanism of action of this extract are now in progress. Potential future research goals include stability tests and the development of formulations.

## Figures and Tables

**Table 1:** Activity of different fractions obtained from *T. diffusa*.

	% recovery	Hepatoprotection, AST (UI/L)	Cytotoxic activity, CC_50_ *μ*g/mL
ME	12.08	95.33 ± 11.1	49.73 ± 5.03
CME	6.04	82.00 ± 6.08	>500
FrVLC	1.98	41.33 ± 6.50	>500
FrMF	0.47	32.33 ± 5.13	>500
Hepatodamianol		20.23 ± 3.67	>500
Silibinin		81.33 ± 6.02	
Doxorubicin			3.12 ± 0.23

Recovery was calculated based on the dry plant. Hepatoprotection model *in vitro*: HepG2 cells; CCl_4_ 0.4% as a damage agent, cells + CCl_4_: AST 111.33 ± 3.51, cells without CCl_4_: AST 0.66 ± 0.57. Cytotoxicity was assessed in Vero cells; *n* = 3.

**Table 2:** Experimental variables used for the optimization of the extraction procedure.

	Low value	Central value	High value
Solvent volume	10 mL	15 mL	25 mL
Stirring time	30 min	60 min	120 min
Stirring speed	100 rpm	200 rpm	300 rpm
Number of extractions	1	3	6

**Table 3:** Parameters of identity and activity for the different HM samples prepared.

Sample	Date of collection	Hepatodamianol content (mg/g sample)	Antioxidant activity (CE_50_ *μ*g/ml)	Flavonoid content (mg equivalent/g sample)		Hepatoprotection activity AST (IU/L)
				Quercetin	Rutin	
HM1	January 2015	16.95 ± 0.31	55.24 ± 7.68	80.11 ± 1.65	186.97 ± 4.01	37.33 ± 5.13
HM2	November 2015	18.42 ± 0.37	62.20 ± 7.97	68.62 ± 0.88	159.09 ± 2.15	38.66 ± 1.15
HM3	November 2014	25.73 ± 0.73	58.74 ± 7.86	52.54 ± 0.66	120.06 ± 1.61	38.66 ± 2.08
HM4	December 2015	15.98 ± 0.34	52.79 ± 6.42	68.18 ± 0.45	153.178 ± 1.10	38.00 ± 2.64
HM5	April 2016	19.33 ± 0.95	78.48 ± 9.09	57.81 ± 0.29	32.86 ± 0.72	33.00 ± 1.00
HM6	July 2015	11.66 ± 1.00	52.13 ± 4.03	88.42 ± 0.52	207.16 ± 1.26	38.33 ± 3.51
HM7	October 2015	13.40 ± 0.34	60.29 ± 8.00	64.68 ± 0.63	149.52 ± 1.53	36.33 ± 5.85
HM8	February 2016	8.76 ± 0.26	66.55 ± 8.69	47.76 ± 0.15	108.45 ± 0.36	39.00 ± 2.64
HM9	June 2015	20.99 ± 0.60	26.92 ± 0.48	162.58 ± 0.36	379.69 ± 0.89	41.33 ± 2.51
HM10	June 2015	7.91 ± 0.25	90.32 ± 1.89	49.36 ± 0.70	112.32 ± 1.69	45.66 ± 3.05
Quercetin			3.88 ± 0.16			
Hepatodamianol			4.76 ± 0.2			20.23 ± 3.67
Silibinin						81.33 ± 6.02

Hepatoprotection model *in vitro*: HepG2 cells, CCl_4_ 0.4% as damage agent, cells + CCl_4_: AST 111.33 ± 3.51, cells without CCl_4_: AST 0.66 ± 0.57. Cytotoxicity was assessed in Vero cells; *n* = 3. HM1 to HM10 are standardized extracts obtained from 10 different samples of *Turnera diffusa* with the procedure described.

## Data Availability

The data used to support the findings of this study are available from the corresponding author upon request.

## Supplementary Materials

Supplementary Materials In the supplementary material, S1 shows the 1HNMR spectrum obtained from hepatodamianol, pointing to the signals corresponding to the methyls in 6´´ and 6´´´positions. In S2, we illustrate the selective 1D-TOCSY experiment for these signals in the isolated compound and the herbal drug to demonstrate the usefulness of this experiment for hepatodamianol identification in complex mixtures and to be used as a spectroscopic fingerprint.

## References

[1] Méndez-Sánchez N., Esquivel M. U. (2003). *Conceptos actuales en hepatología*.

[2] Hiraganahalli B. D., Chinampudur V. C., Dethe S. (2012). Hepatoprotective and antioxidant activity of standardized herbal extracts. *Pharmacognosy Magazine*.

[3] Asrani S. K., Devarbhavi H., Eaton J., Kamath P. S. (2019). Burden of liver diseases in the world. *Journal of Hepatology*.

[4] Tsague M. K., Bomgning L. C., Fofié C. K., Nguelefack-Mbuyo E. P., Fotio A. L., Nguelefack T. B. (2020). Hepatoprotective effects of the leaves of *Agauria salicifolia* against acetaminophen-induced liver injury in mice. *Journal of Biosciences and Medicines*.

[5] Ielciu I., Sevastre B., Olah N. K. (2021). Evaluation of hepatoprotective activity and oxidative stress reduction of *Rosmarinus officinalis* L. shoots tincture in rats with experimentally induced hepatotoxicity. *Molecules*.

[6] Farghali H., Canová N. K., Zakhari S. (2015). Hepatoprotective properties of extensively studied medicinal plant active constituents: possible common mechanisms. *Pharmaceutical Biology*.

[7] OMS (2014). *OMS-Cirrosis*.

[8] INEGI (2019). *Características de las Defunciones Registradad en México durante 2018*.

[9] Newman D. J., Cragg G. M. (2020). Natural products as sources of new drugs over the nearly four decades from 01/1981 to 09/2019. *Journal of Natural Products*.

[10] Torres González L., Waksman de Torres N., Pérez Meseguer J., Muñoz Espinosa L. E., Salazar Aranda R., Cordero Pérez P. (2014). Review of plants with hepatoprotective activity evaluated in México. *Medicina Universitaria*.

[11] Torres-González L., Muñoz-Espinosa L. E., Rivas-Estilla A. M. (2016). Protective effect of four Mexican plants against CCl4-induced damage on the Huh7 human hepatoma cell line. *Annals of Hepatology*.

[12] Delgado-Montemayor C., Pérez-Meseguer J., Salazar-Aranda R., Cordero-Perez P., Torres-González L., Waksman N. (2019). *México. Patente MX/a/2014/008805*.

[13] Zhao J., Pawar R. S., Ali Z., Khan I. A. (2007). Phytochemical investigation of *Turnera diffusa*. *Journal of Natural Products*.

[14] Rodríguez-Rodríguez D. R., Lozano-Sepulveda S. A., Delgado-Montemayor C., Waksman N., Cordero-Perez P., Rivas-Estilla A. M. (2021). *Turnera diffusa* extract attenuates profibrotic, extracellular matrix and mitochondrial markers in activated human hepatic stellate cells (HSC). *Annals of Hepatology*.

[15] Mosmann T. (1983). Rapid colorimetric assay for cellular growth and survival: application to proliferation and cytotoxicity assays. *Journal of Immunological Methods*.

[16] González L. T., Minsky N. W., Espinosa L. E., Aranda R. S., Meseguer J. P., Pérez P. C. (2017). In vitro assessment of hepatoprotective agents against damage induced by acetaminophen and CCl4. *BMC Complementary and Alternative Medicine*.

[17] Granados-Guzmán G., Salazar-Aranda R., Garza-Tapia M., Castro-Ríos R., Waksman de Torres N. (2017). Optimization and validation of two high-throughput methods indicating antiradical activity. *Current Analytical Chemistry*.

[18] Kumazawa S., Hamasaka T., Nakayama T. (2004). Antioxidant activity of propolis of various geographic origins. *Food Chemistry*.

[19] USP 32-NF 27 (2007). *General Chapter USP Organic Volatile Impurities, United States Pharmacopeia*.

[20] Lucio-Gutiérrez J. R., Delgado-Montemayor C., Coello-Bonilla J., Waksman-Minsky N., Saucedo A. L. (2019). Selective 1D-TOCSY and chemometrics to evaluate authenticity of Turnera diffusa and related botanical extracts. *Phytochemistry Letters*.

[21] Cordero-Pérez P., Torres-González L., Aguirre-Garza M. (2013). Hepatoprotective effect of commercial herbal extracts on carbon tetrachloride-induced liver damage in Wistar rats. *Pharmacognosy Research*.

[22] Avelino-Flores M. D. C., Cruz-López M. D. C., Jiménez-Montejo F. E., Reyes-Leyva J. (2015). Cytotoxic activity of the methanolic extract of *Turnera diffusa* Willd on breast cancer cells. *Journal of Medicinal Food*.

[23] Willer J., Jöhrer K., Greil R., Zidorn C., Çiçek S. S. (2019). Cytotoxic properties of Damiana (*Turnera diffusa*) extracts and constituents and a validated quantitative UHPLC-DAD assay. *Molecules*.

[24] Haddad Y., Vallerand D., Brault A., Haddad P. S. (2011). Antioxidant and hepatoprotective effects of silibinin in a rat model of nonalcoholic steatohepatitis. *Evidence-based Complementary and Alternative Medicine*.

[25] Pérez-Meseguer J., Garza-Juárez A., Salazar-Aranda R. (2010). Development and validation of an HPLC-DAD analytical procedure for quality control of damiana (*Turnera diffusa*), using an antioxidant marker isolated from the plant. *Journal of AOAC International*.

[26] Papaemmanouil C., Tsiafoulis C. G., Alivertis D. (2015). Selective one-dimensional total correlation spectroscopy nuclear magnetic resonance experiments for a rapid identification of minor components in the lipid fraction of milk and dairy products: toward spin chromatography?. *Journal of Agricultural and Food Chemistry*.

[27] Sandusky P., Raftery D. (2005). Use of selective TOCSY NMR experiments for quantifying minor components in complex mixtures: application to the metabonomics of amino acids in honey. *Analytical Chemistry*.

[28] Brito N. J. N., López J. A., Nascimento M. A. d. (2012). Antioxidant activity and protective effect of *Turnera ulmifolia* linn. var. elegans against carbon tetrachloride-induced oxidative damage in rats. *Food and Chemical Toxicology*.

