# Supplementary material for: Development of a Hepatoprotective Herbal Drug from Turnera diffusa
Source: Evid Based Complement Alternat Med. 2022 Jan 10;2022:5114948. doi: 10.1155/2022/5114948 (PMC8763504; doi:10.1155/2022/5114948)
Supplement: Supplementary Materials — In the supplementary material, S1 shows the 1HNMR spectrum obtained from hepatodamianol, pointing to the signals corresponding to the methyls in 6´´ and 6´´´positions. In S2, we illustrate the selective 1D-TOCSY experiment for these signals in the isolated compound and the herbal drug to demonstrate the usefulness of this experiment for hepatodamianol identification in complex mixtures and to be used as a spectroscopic fingerprint. [file 5114948.f1.docx]

Supplementary material


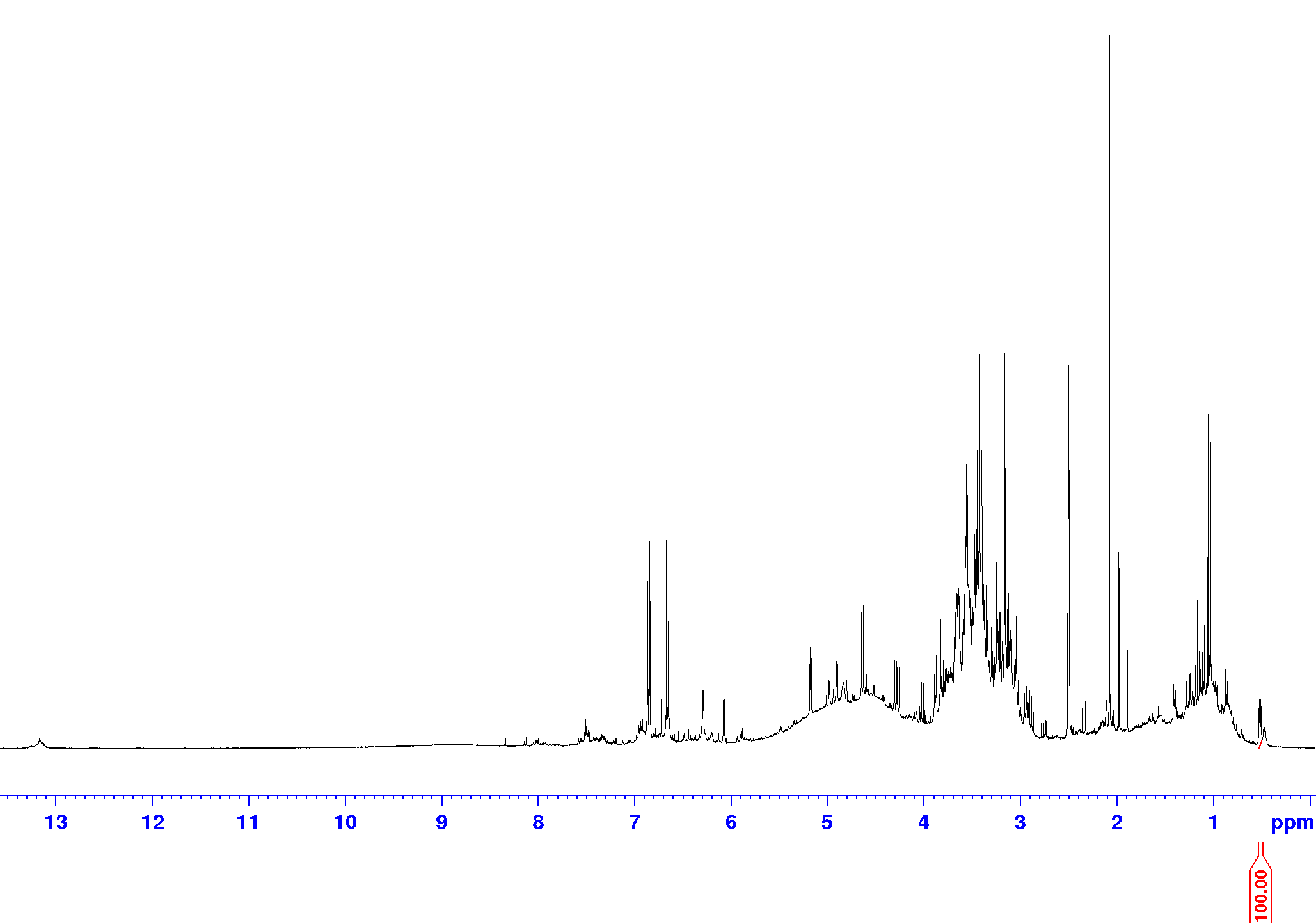


6´´

6´´´


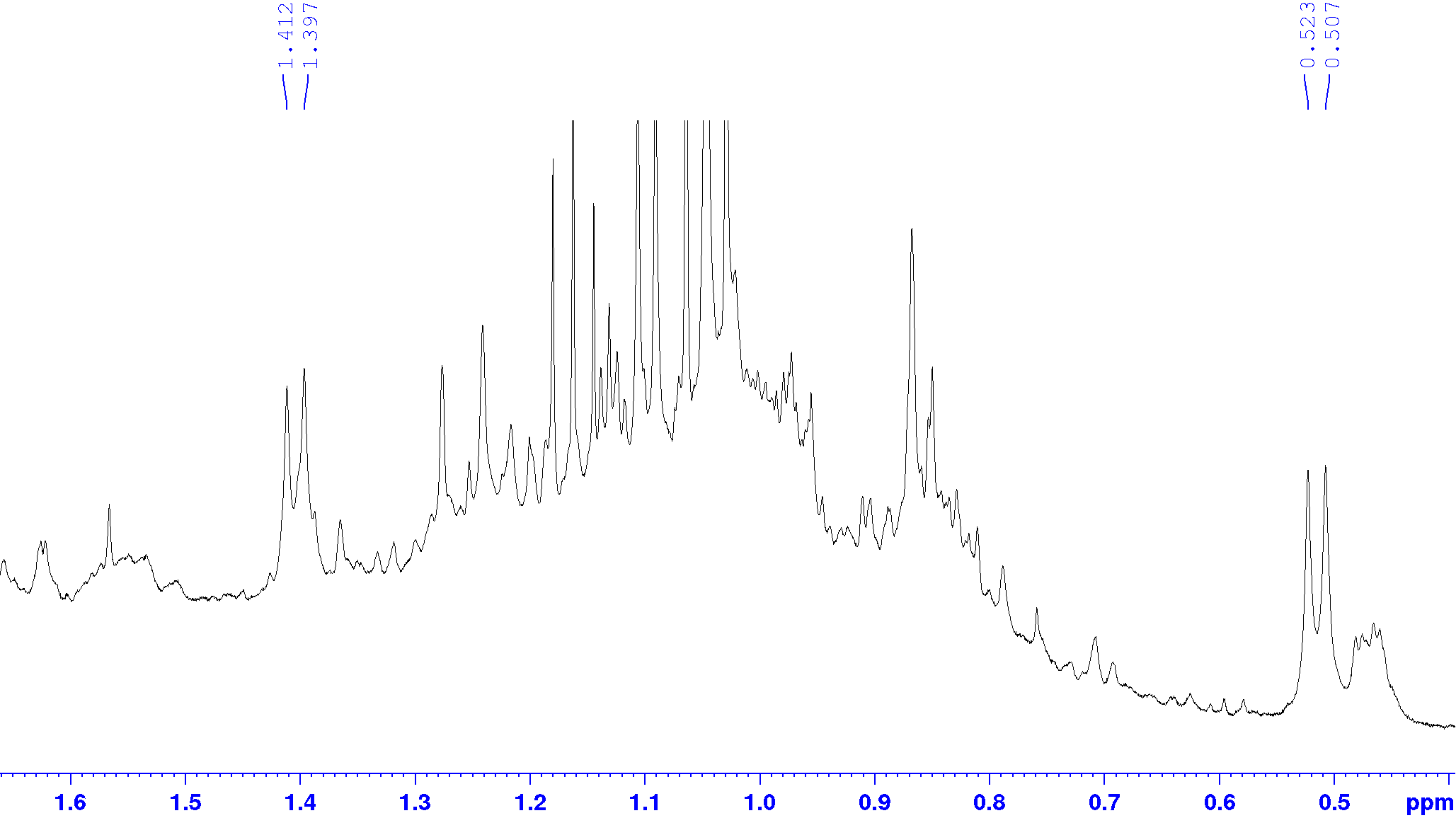


6´´´

6´´

S1. ^1^HNMR spectrum of hepatodamianol, 400 MHz, DMSO-*d*6

**A**

**B**

**C**

**D**


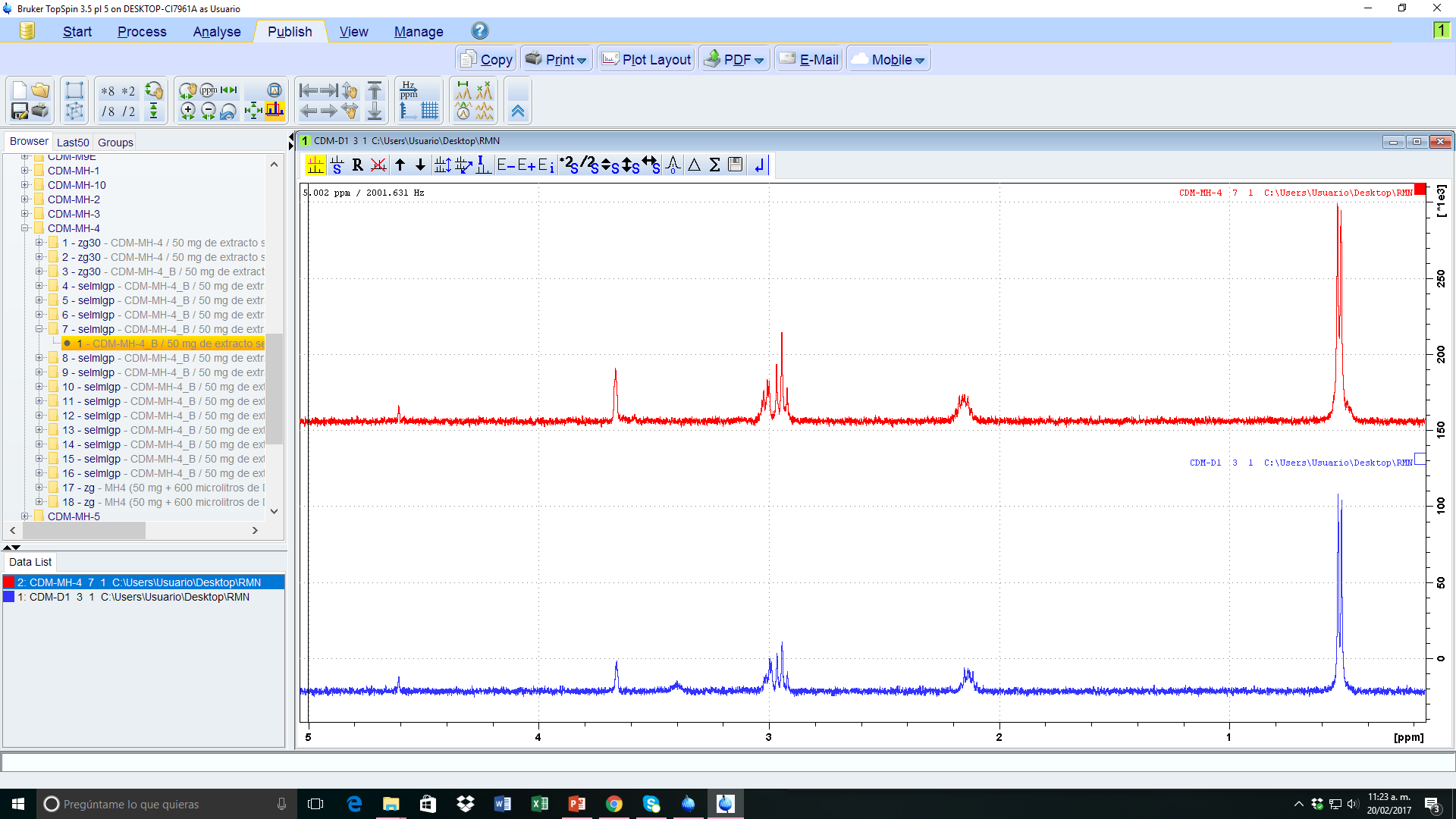


Hepatodamianol

HM4


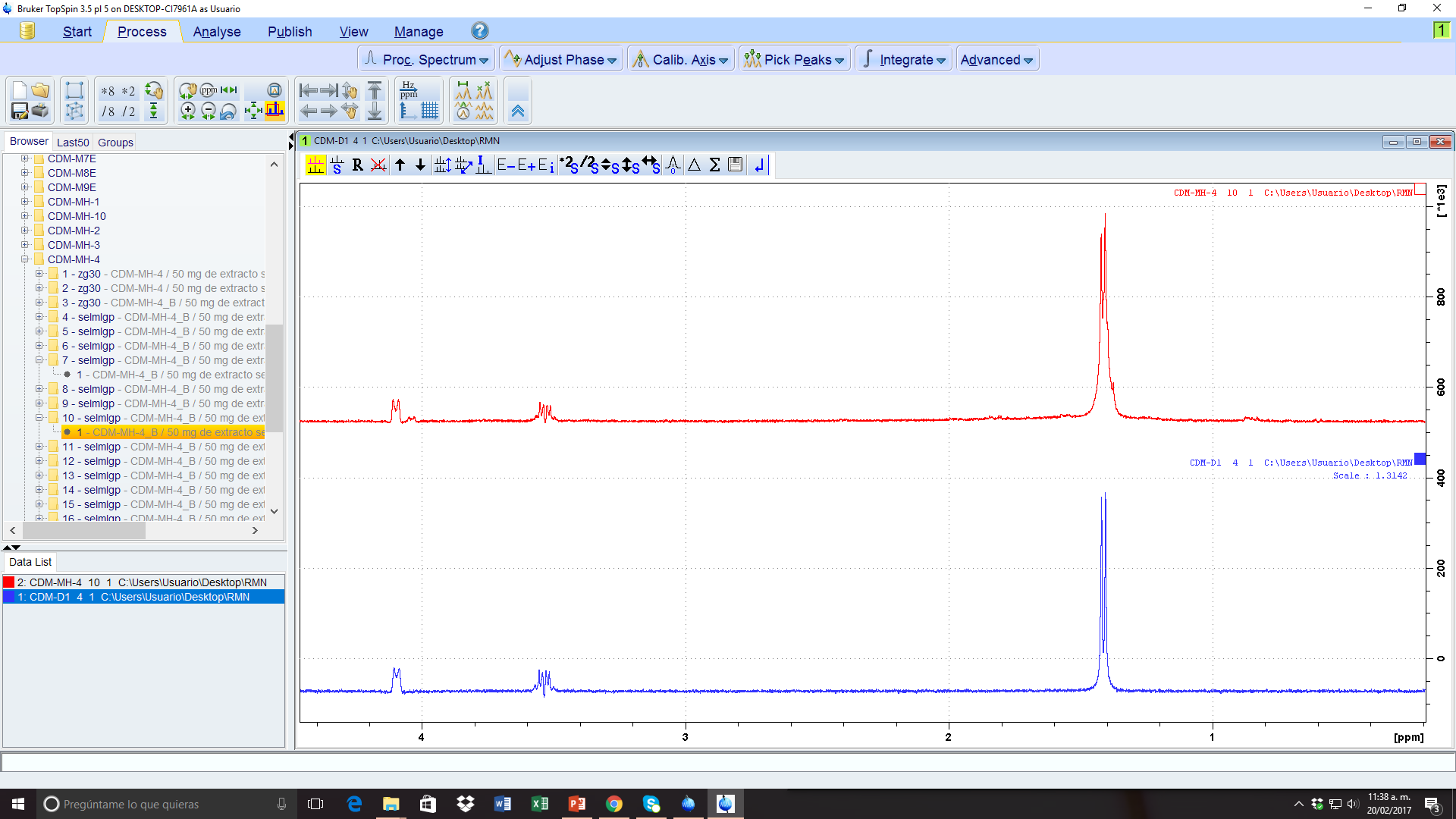


O1= 1.40 ppm (564 Hz)

τm= 50 ms

4´´

5´´

HM4

Hepatodamianol

4´´

5´´

6´´

6´´

6´´´

6´´´

5´´´

5´´´

1´´´

3´´´ 4´´´

2´´´

3´´´ 4´´´

2´´´

1´´´

O1= 0.515 ppm (219 Hz)

τm= 200 ms

S2. **A**. Selective 1D-TOCSY of methyl 6′′ (1.40 ppm) in hepatodamianol rhamnopyranosyl motif. **B**. Selective 1D-TOCSY of HM4 with irradiation on 1.40 ppm and identification of hepatodamianol rhamnopyranpsyl motif. **C**. Selective 1D-TOCSY of methyl 6′´′ (0.515 ppm) in hepatodamianol hexopyran-3-uloside motif. **D**. 1H-NMR spectrum of HM4 with irradiation on 0.515 ppm and identification of hepatodamianol hexopyran-3-uloside motif.

Hepatodamianol

MH4

6´´

6´´
